# Kaposi’s Sarcoma-Associated Herpesvirus (KSHV) Induces the Oncogenic miR-17-92 Cluster and Down-Regulates TGF-β Signaling

**DOI:** 10.1371/journal.ppat.1005255

**Published:** 2015-11-06

**Authors:** Hong Seok Choi, Vaibhav Jain, Brian Krueger, Vickie Marshall, Chang Hee Kim, Joanna L. Shisler, Denise Whitby, Rolf Renne

**Affiliations:** 1 Department of Molecular Genetics and Microbiology, University of Florida, Gainesville, Florida, United States of America; 2 AIDS and Cancer Virus Program, Leidos Biomedical, Frederick National Laboratory for Cancer Research, Frederick, Maryland, United States of America; 3 Department of Microbiology, College of Medicine, University of Illinois, Urbana, Illinois, United States of America; 4 UF Health Cancer Center, University of Florida, Gainesville, Florida, United States of America; 5 UF Institute of Genetics, University of Florida, Gainesville, Florida, United States of America; University of North Carolina at Chapel Hill, UNITED STATES

## Abstract

KSHV is a DNA tumor virus that causes Kaposi’s sarcoma. Upon KSHV infection, only a limited number of latent genes are expressed. We know that KSHV infection regulates host gene expression, and hypothesized that latent genes also modulate the expression of host miRNAs. Aberrant miRNA expression contributes to the development of many types of cancer. Array-based miRNA profiling revealed that all six miRNAs of the oncogenic miR-17-92 cluster are up-regulated in KSHV infected endothelial cells. Among candidate KSHV latent genes, we found that vFLIP and vCyclin were shown to activate the miR-17-92 promoter, using luciferase assay and western blot analysis. The miR-17-92 cluster was previously shown to target TGF-β signaling. We demonstrate that vFLIP and vCyclin induce the expression of the miR-17-92 cluster to strongly inhibit the TGF-β signaling pathway by down-regulating SMAD2. Moreover, TGF-β activity and SMAD2 expression were fully restored when antagomirs (inhibitors) of miR-17-92 cluster were transfected into cells expressing either vFLIP or vCyclin. In addition, we utilized viral genetics to produce vFLIP or vCyclin knock-out viruses, and studied the effects in infected TIVE cells. Infection with wildtype KSHV abolished expression of SMAD2 protein in these endothelial cells. While single-knockout mutants still showed a marked reduction in SMAD2 expression, TIVE cells infected by a double-knockout mutant virus were fully restored for SMAD2 expression, compared to non-infected TIVE cells. Expression of either vFLIP or vCycIin was sufficient to downregulate SMAD2. In summary, our data demonstrate that vFLIP and vCyclin induce the oncogenic miR-17-92 cluster in endothelial cells and thereby interfere with the TGF-β signaling pathway. Manipulation of the TGF-β pathway via host miRNAs represents a novel mechanism that may be important for KSHV tumorigenesis and angiogenesis, a hallmark of KS.

## Author Summary

MiRNAs are small non-coding RNAs which decrease gene expression and function as oncogenes or tumor suppressors. Dysregulation of miRNAs is a hallmark of many human cancers. Recently, it was revealed that the miR-17-92 cluster, up-regulated in many cancers, plays a central role in down-regulation of the TGF-β signaling pathway. Kaposi’s sarcoma-associated herpesvirus (KSHV) is a gamma-herpesvirus associated with Kaposi’s sarcoma and two lymphoproliferative diseases. KSHV is known to target the TGF-β pathway. Here, we found that two viral latent genes, vFLIP and vCyclin, blunt TGF-β signaling by inducing the host miR-17-92 cluster. Moreover, we confirmed that endothelial cells infected with wt KSHV gave no expression of SMAD2, a key component in the TGF-β pathway. Using a vFLIP vCyclin double knock-out mutant virus gave complete restoration of SMAD2 expression in endothelial cells. This finding reveals a new pathway that KSHV utilizes to promote tumorigenesis and angiogenesis in Kaposi’s sarcoma.

## Introduction

Kaposi’s sarcoma-associated herpes virus (KSHV) is a member of the gammaherpesvirus family and is associated with Kaposi’s sarcoma (KS), a subset of multicentric Castleman’s disease (MCD), and primary effusion lymphoma (PEL) [1,2,3]. KSHV has two distinct phases of infection, latent and lytic. During latency, a small subset of KSHV genes is expressed from the KSHV latency associated region (KLAR). Latent gene products including kaposin, viral Fas-associated death domain IL-1β-converting enzyme inhibitory protein (vFLIP), viral cyclin (vCyc), latency-associated nuclear antigen (LANA), and viral micro RNAs (miRNAs), contribute to the survival and proliferation of KSHV- infected tumor cells.

Viral FLIP is a homolog of cellular FLIP, which can protect cells from Fas-mediated apoptosis. Furthermore, vFLIP does not just block the extrinsic signal but also induces NF-κB signaling, which is important for viral latency and tumorigenesis [4]. Rat-1 cells expressing vFLIP promote the tumor formation in nude mouse, which is associated with NF-κB activation [5]. While vFLIP is a potent inducer of apoptosis and required for PEL cell survival its expression levels are tightly regulated in part due to usage of rare codons [6]. Inducible expression of vFLIP alone in mouse endothelial cells leads to induction of serum proinflammatory cytokines *in vivo*, and alterations in myeloid differentiation [7]. In addition to regulating apoptosis and autophagy [8], and activating NF-κB, vFLIP proteins also regulate intrinsic innate immunity by targeting IRF-3 (reviewed in [9]). The viral cyclin (vCyc) is a homolog of cellular cyclin D, which functions at the G1/S cell cycle transition by activation of cyclin dependent kinase 6 (cdk6). Unlike cellular cyclin D, vCyc is resistant to p27 cdk inhibitor [10,11]. Despite resistance to cdk inhibitors, the activity of KSHV cyclin is blocked by p53. However, it is reported that vCyc is sufficient to promote proliferation of latently infected cells thereby potentially contributing to cell transformation and tumorigenesis in the presence of p53 inhibiting factors, such as LANA [12].

MicroRNAs are short (21~25 nucleotides) noncoding RNAs that down-regulate gene expression post-transcriptionally by binding to the 3’-untranslated region (3’-UTR) of target messenger RNA (mRNA) with partial complementarity [13]. miRNAs play a central role in central biological processes, such as development, differentiation, apoptosis, and proliferation [14]. Dysregulation of miRNAs is not only a hallmark of many human malignancies but is also involved in the development and progression of cancer. MiRNAs either target tumor suppressor genes or by themselves can have oncomir or tumor promoting activity (for review see [15]).

The miR-17-92 cluster is one of the well-characterized oncogenic miRNA clusters, for which aberrant expression was found in various types of cancers and which has been shown to play a critical role in development. miR-17, 18a, 19a, 20a, 19b-1, and 92a-1, the members of the miR-17-92 cluster, are derived from a single polycistronic transcript located at chromosome 13q31. Amplification of this region is found in several types of lymphomas and lung cancer and overexpression in transgenic mice causes B cell lymphomas [16,17]. The miR-17-92 cluster is regulated by the transcription factor, c-Myc, that is frequently hyperactive in many types of cancers. With respect to cell cycle control, the miR-17-92 cluster miRNAs target the E2F transcription factor family but are also activated by E2F, thereby establishing a negative feedback loop [18,19,20]. In neuroblastoma cells, miR-17-92 has been shown to target components of the TGF-β signaling pathway. TGF-β receptor 2 is targeted by miR-17 and miR-20, and SMAD2 and SMAD4 are inhibited by miR-18a [21]. In addition, two homologous clusters, miR106a-363 and miR106b-25, are located at the X and chromosome 7, respectively. The 15 miRNAs expressed from these three clusters represent 4 different seed sequence families which are miR-17, 18, 19, and 92 [22].

The TGF-β signaling pathway is involved in many cellular events, but in the context of KSHV pathogenesis, the most relevant functions of TGF-β are suppression of cell growth and promotion of apoptosis [23]. The importance of TGF-β during latent KSHV infection is supported by the fact that KSHV negatively modulates TGF-β signaling by different mechanisms. Multiple KSHV miRNAs inhibit TGF-β maturation by targeting thrombospondin1 (THBS1) [24]. KSHV LANA targets the TGF-β pathway by reducing the expression of TGF-β receptor type 2 (TGBR2) [25]. TGF-β signaling in developing or progressing cancers is highly context-dependent, since TGF-β signaling has both pro-apoptotic and proliferative properties [26]. It is widely thought that inhibition of TGF-β signaling is important during the early stages of tumorigenesis, even though the pathway is subsequently reactivated in the later stages of cancers associated with metastasis. TGF-β can also block cell cycle progression by inducing the expression of cdk inhibitors [27,28].

We performed miRNA profiling and observed increased expression of the tumorigenic miR-17-92 cluster in KSHV latently infected cells. Testing of KSHV latency associated genes for their ability to up-regulate the miR-17-92 cluster revealed that vFLIP and vCyclin augment transcription from the miR-17-92 promoter which in turn strongly down-regulates TGF-β signaling.

## Results

### The oncogenic miR-17-92 cluster is up-regulated in KSHV latently infected cells

**Fig 1:**
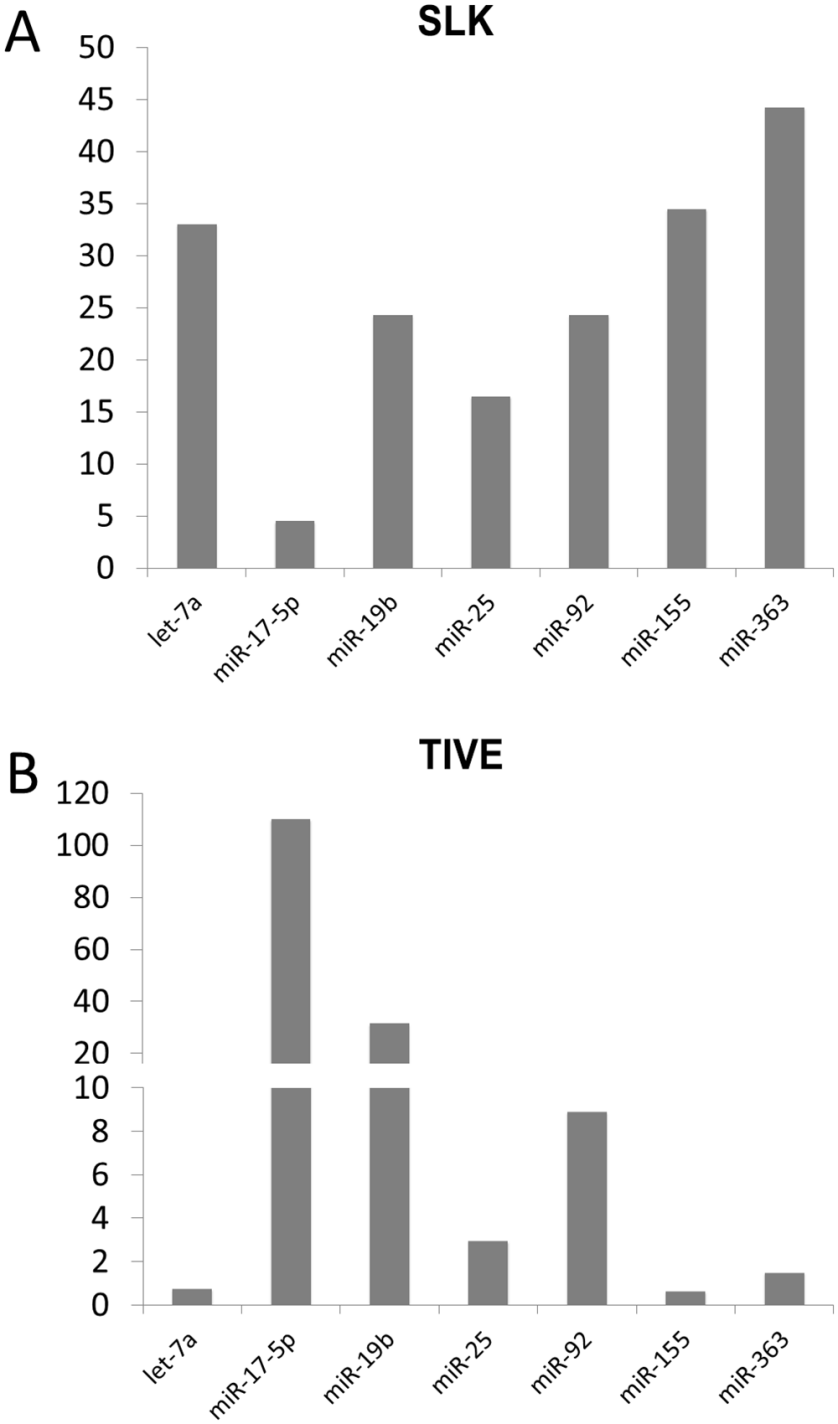
Quantitative RT-PCR analysis of miRNAs in latently KSHV-infected SLK and TIVE cells. The expression differences of miRNAs in KSHV-infected SLK (A) and TIVE cells (B) from non-infected SLK and TIVE cells. U6 expression was used as internal control for normalization.

KSHV encodes 12 miRNA genes and in latently infected cells viral miRNAs can represent a significant percentage of all miRNAs within active RISCs [29]. Additionally, previous reports have analyzed host cellular miRNA expression patterns in KSHV-infected tumor cells [30]. Hence, we hypothesized that infection with KSHV not only modulates host cellular gene expression through viral miRNA targeting but also by directly perturbing cellular miRNA expression. To test this hypothesis, we performed microarray-based miRNA profiling of host miRNAs in mock or stably KSHV-infected SLK and long-term infected TIVE-LTC cells. A custom made array was designed that contained probes for a total of 1667 human miRNAs and several control genes for normalization (for details see Materials and Methods). In KSHV-infected SLK cells, which are of epithelial origin, 80 miRNAs were up-regulated 2-fold or more, compared to mock. Among the highest induced were the oncomir miR-155, miR-27, and several members of the let-7 family (Fig 1A and Table 1). Comparison of TIVE to TIVE/LTC, which are of endothelial origin, revealed 103 miRNAs that were up-regulated 2-fold or more and 8 miRNAs that were down-regulated including miR-125 and miR-100. The top eight induced host miRNAs represented members of the oncogenic miR-17-92 cluster and its orthologs. Comparison of both data sets revealed 59 miRNAs including the miR-17-92 cluster that were commonly up-regulated in both KSHV-infected TIVE and SLK cells. Additional miRNAs that are known to be aberrantly expressed in various cancers and were induced in both cell lines included the let-7 family, miR-16, miR-21, and miR-34 (Fig 1B and Table 1).

**Table 1:** Expression of miRNAs in uninfected and KSHV-infected SLK and TIVE cells.

miR	SLK	SLK KS	Fold increase SLK KS/SLK	TIVE	TIVE-LTC	Fold increase TIVE-LTC/TIVE
17	1979	6761	3.4	169*	15443	IND^1^
18a	635	1545	2.4	82*	5155	IND^1^
19a	1581	6555	4.1	124*	23760	IND^1^
20a	1642	5369	3.3	117*	14502	IND^1^
19b^2^	2436	12798	5.3	252*	32731	IND^1^
92^3^	311	1158	3.7	159*	1615	IND^1^
106a	1833	6475	3.5	139*	14959	IND^1^
18b	481	1221	2.5	61*	4038	IND^1^
20b	918	3365	3.7	85*	9043	IND^1^
363	91*	489	IND^1^	374	1795	4.7
106b	334	1612	4.8	181*	3140	IND^1^
93	134*	915	IND^1^	131*	1548	IND^1^
25	177*	641	IND^1^	208*	1180	IND^1^
155	146*	1833	IND^1^	600	2509	4.2

*below the detection threshold

^1^ IND: induced but fold increase cannot be calculated as expression level in uninfected cells is below the detection threshold

^2^ Probes for miR-19b detect mature products (miR-19b-3p) of both miR-19b-1 (chr 13) and miR-19b-2 (Chr X).

^3^ Probes for miR-92 detect mature products (miR-92a-3p) of both miR-92a-1 (chr 13) and miR-92a-2 (Chr X).

Since the miR-17-92 cluster has known oncomir activity and like the miR-155 pathway is targeted in many human malignancies including PEL [31,32], we focused on the regulation of these miRNAs (Fig 1 and Table 1). We note that in TIVE cells, the miR-17-92 cluster miRNAs are not detectable, while they are highly expressed in TIVE-LTC. To confirm the array analysis the expression levels of seven miRNAs were analyzed by stem-loop qRT-PCR assays. As shown in Fig 1 and Table 1, up-regulation of three miR-17-92 cluster miRNAs (mir-17-5p, mir-19b, and miR-92) were confirmed for both KSHV-infected SLK and TIVE cells.

### The miR-17-92 cluster is transcriptionally up-regulated by vFLIP and vCyclin

**Fig 2:**
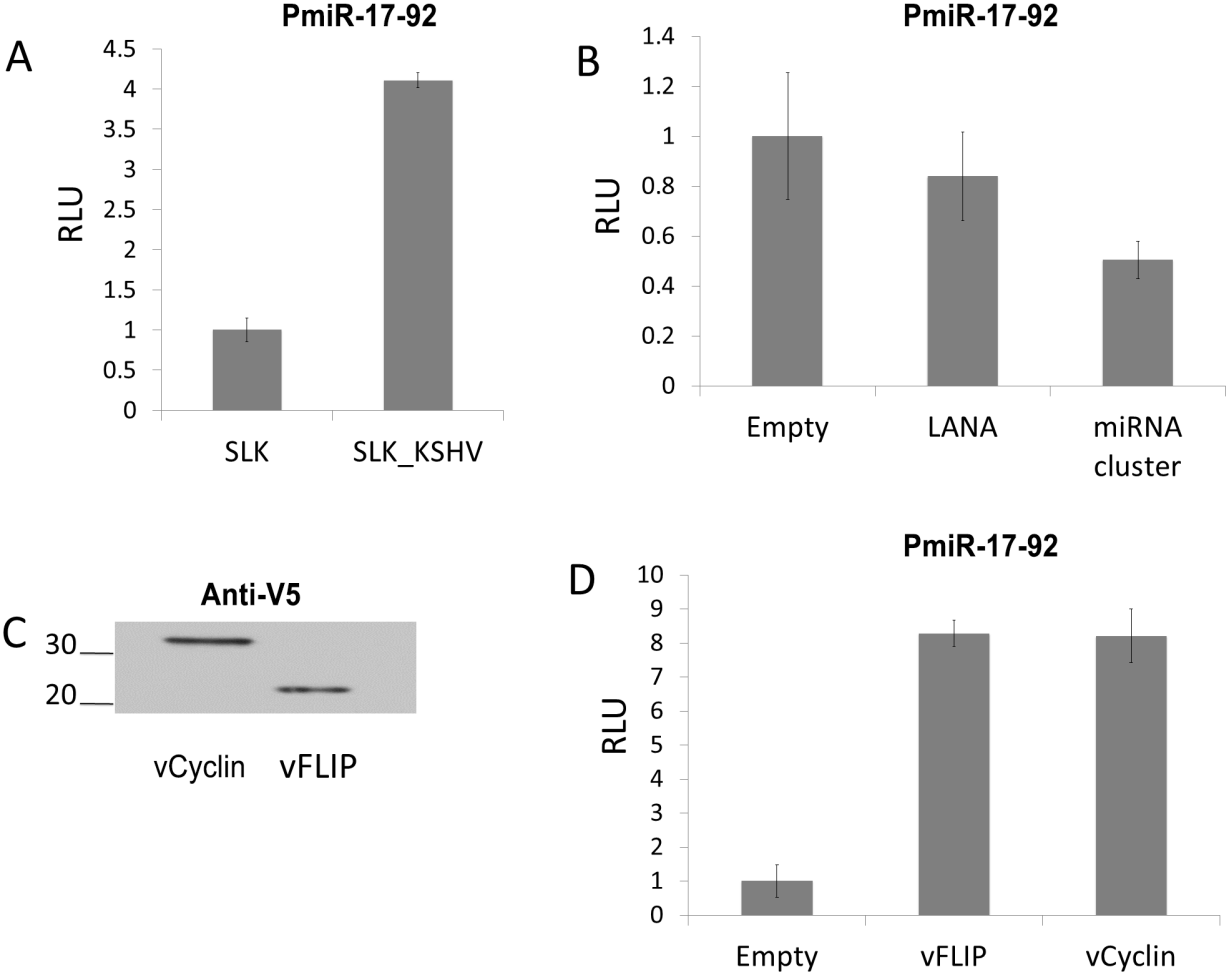
KSHV infection increases expression from the miR-17-92 cluster promoter via vFLIP and vCyclin. (A) Uninfected SLK cells and SLK cells latently infected with KSHV were electroporated with reporter vector (pGL3-PmiR-17-92) containing the promoter of the miR-17-92 cluster upstream of luciferase gene [19]. The firefly luciferase activity was measured then set at 1 for uninfected SLK cells. Error bars show standard deviation for triplicate experiments. (B) Co-transfection of pGL3-PmiR-17-92 with plasmids expressing LANA or the KSHV miRNA cluster. pGL3-PmiR-17-92 was co-transfected with empty vector or with pcDNA3/LANA or pcDNA3.1/cluster into SLK cells using Mirus293IT transfection reagent. Firefly and Renilla luciferase activities were measured, and firefly luciferase activity was normalized to control Renilla luciferase activity. Activity was set at 1 for cells transfected with empty vector. (C) Confirmation of expression of V5-tagged vFLIP and vCyclin by Western blotting. Extracts of cells transfected with pLenti6/vFLIP or pLenti6/vCyc were probed with anti-V5 mAb. D) Co-transfection of pGL3-PmiR-17-92 with plasmids expressing vFLIP or vCyclin. pLenti6/vFLIP or pLenti6/vCyc were co-transfected with reporter vector into SLK cells and firefly luciferase activity measured and normalized as above.

To determine whether the observed up-regulation of the miR-17-92 cluster is due to transcriptional regulation a reporter assay was performed. A vector, containing the promoter of the miR-17-92 cluster upstream of the luciferase gene, was transfected into mock or latently KSHV-infected SLK cells. The miR-17-92 promoter showed approximately 4-fold increased activity in KSHV-infected SLK cells compared to uninfected cells (Fig 2A). Since KSHV infected SLK cells express only a limited subset of genes during latency these results suggested that latent KSHV genes contribute to augmenting expression of the miR-17-92 cluster. Thus, we investigated which latent genes are responsible for increasing miR-17-92 expression by testing LANA, the miRNA cluster, vFLIP and vCyclin. LANA and the KSHV miRNA cluster expression plasmids have previously been described [24,33]. Tagged vFLIP and vCyclin were cloned into the Gateway vector pLenti6-V5 (pLenti6/vFLIP and pLenti6/vCyc) and expression was confirmed by Western blot analysis (Fig 2C). Next, expression vectors were co-transfected with the miR-17-92 promoter luciferase reporter. While LANA and viral miRNA expression did not affect luciferase expression from the miR-17-92 promoter (Fig 2B), vFLIP and vCyclin each increased luciferase expression by a factor of 8 (Fig 2D). Hence, these data show that two latency-associated genes vFLIP and vCyclin are responsible for the up-regulation of the miR-17-92 cluster. Next we investigated the consequences of up-regulation of the miR-17-92 cluster.

### TGF-β signaling is down-regulated by vFLIP and vCyclin via increased expression of the miR-17-92 cluster

**Fig 3:**
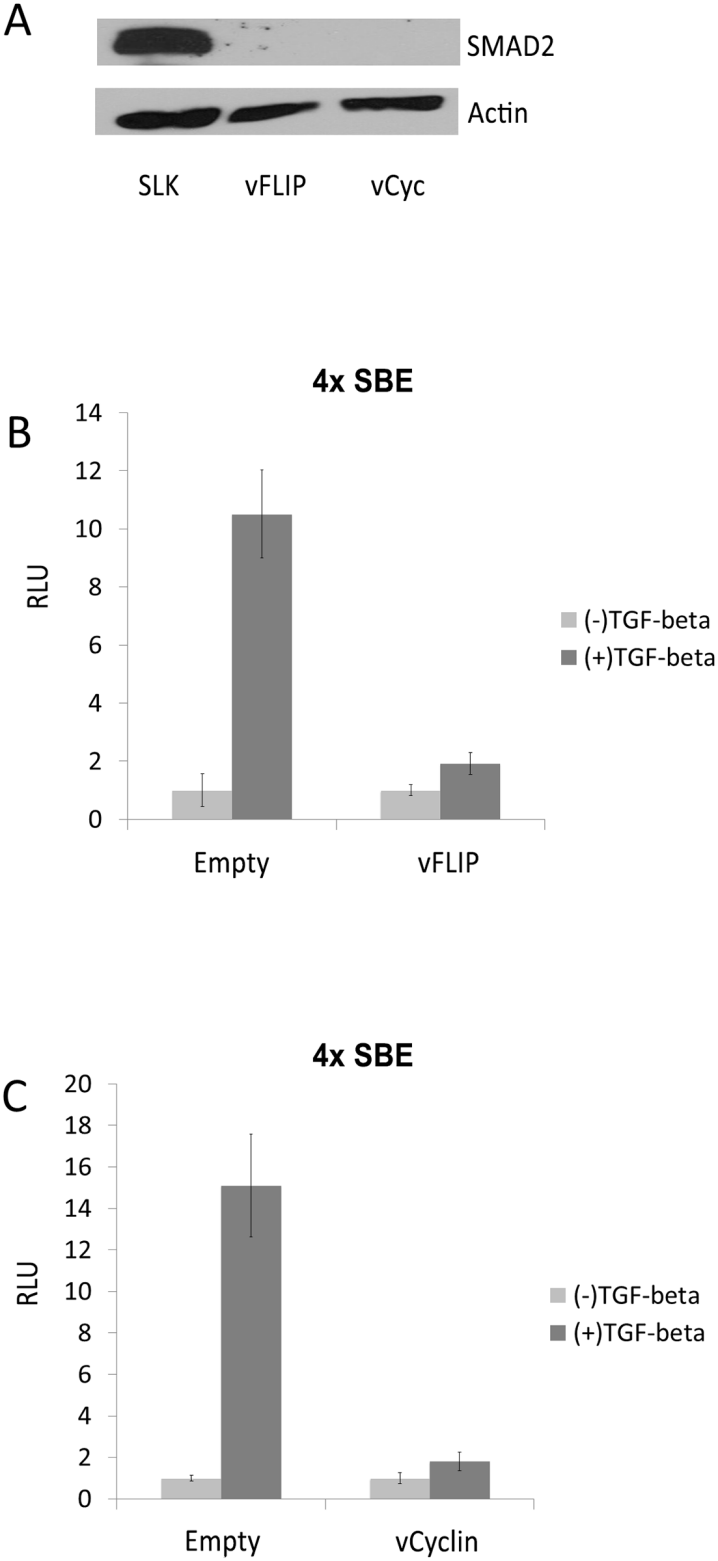
vFLIP and vCyclin decrease SMAD2 protein expression and desensitize TGF-β signaling. (A) SMAD2 expression was drastically decreased upon the expression of vFLIP or vCyclin in SLK cells. Western blot for SMAD2 was performed with SLK cells transfected with empty vector or with vFLIP and vCyclin expression vectors. Actin was detected as internal control. (B and C) vFLIP (B) and vCyclin(C) diminished the response of SLK cells to TGF-β ligand. Luciferase reporter analysis was carried out with pGL3-SBE4, which contains 4 SMAD-binding elements, co-transfected with empty vector or with vectors expressing vFLIP or vCyclin in the absence or presence of TGF-β ligand. TGF-β ligand was added 24 hrs after transfection and the cells were harvested 72 hrs after transfection. The luciferase activities in the presence of TGF-β were normalized with the activities in the absence of TGF-β.

**Fig 4:**
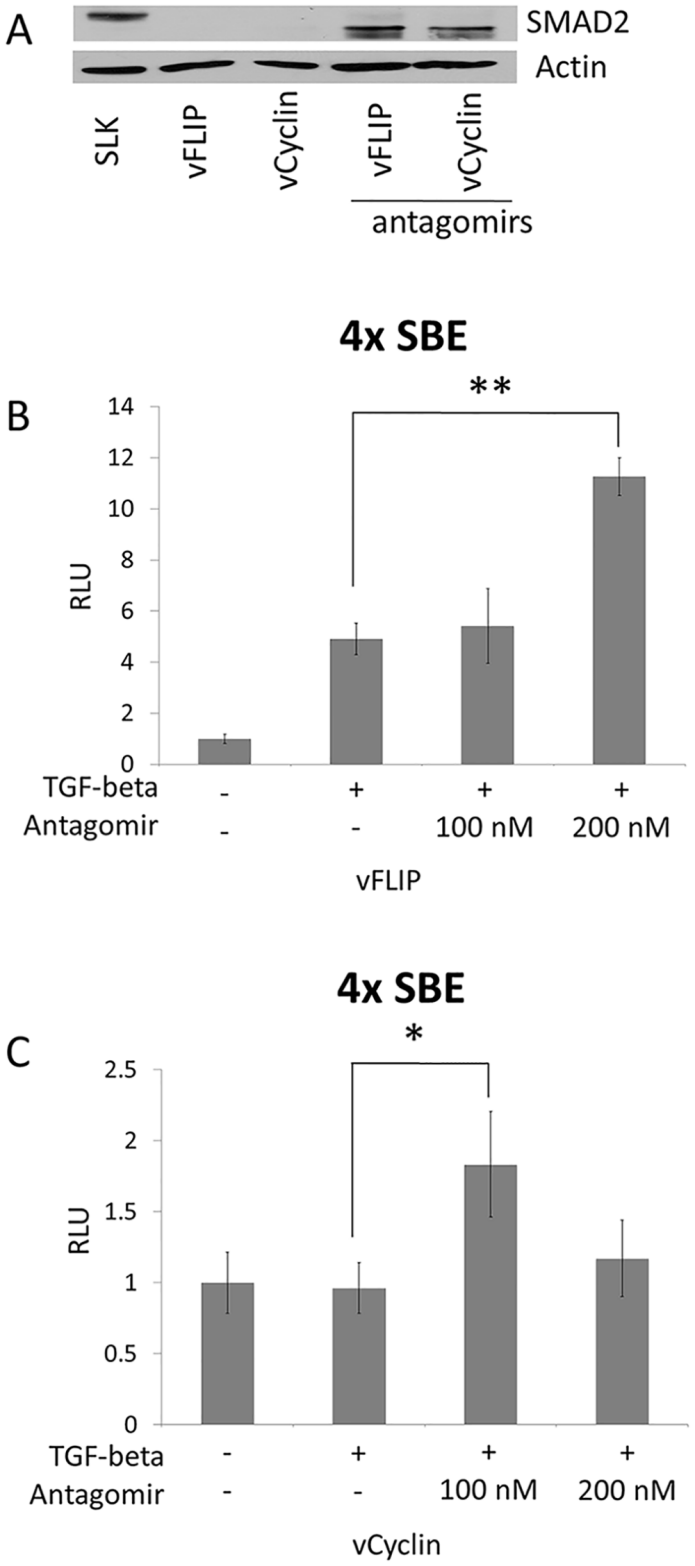
SMAD2 expression and the response to TGF-β were restored by antagomir against the miR-17, 18a, and 20. (A) Western blot for SMAD2 after blocking miR-17-92 cluster. Empty vector or vFLIP or vCyclin expression vectors were transfected into SLK cells with or without 100 nM of antagomirs against miR17, 18a, and 20. Actin was used as internal control. (B and C) Reporter vector (pGL3-SBE4) was co-transfected into SLK cells with vFLIP (B) or vCyclin (C) expression vectors in the absence or presence of antagomirs at 100 nM or 200 nM as indicated. TGF-β ligand was added and the cells were harvested as described in Fig 3. Luciferase activity was measured and set at 1 for cells without TGF-β. * and ** indicate p < 0.05 and p<0.01, respectively, in comparison with cells not treated with antagomir.

It was previously demonstrated that three miRNAs of the miR-17-92 cluster target SMAD2 in neuroblastoma cells. TGF-β is a cytokine with anti-proliferative and pro-apoptotic effects, and this important pathway is known to be targeted by KSHV miRNAs and LANA [24,25]. Thus, we asked if the miR-17-92 cluster up-regulated by vFLIP and vCyclin caused decreased SMAD2 expression. We performed Western blot assay with lysates of SLK cells ectopically expressing vFLIP or vCyclin. Surprisingly, the expression of SMAD2 protein, which is readily detectable in SLK cells, was completely abolished by expression of either vFLIP or vCyclin (Fig 3A). In order to check if TGF-β signaling via SMAD2 is disrupted by vFLIP or vCyclin expression, we performed a luciferase reporter assay with a plasmid containing 4 SMAD binding elements upstream from luciferase. Luciferase activity was induced more than 10-fold in the presence of TGF-β ligand, demonstrating that SLK cells are sensitive to TGF-β treatment. However, transfection of vFLIP or vCyclin diminished the response to the TGF-β measured by luciferase activity (Fig 3B and 3C). Although TGF-β signaling is disrupted by vFLIP and vCyclin expression, it was unclear if vFLIP and vCyclin down-regulate SMAD2 by stimulating miR-17-92 expression. To address this question we co-transfected vFLIP or vCyc expression vectors with sequence-specific antagomirs against miR-17-5p, 18a, and 20 to inhibit the miR-17-92 function, and performed a SMAD2 Western blot. In Fig 4A, SMAD2 expression inhibited by vFLIP and vCyclin was restored in the presence of miR-17-92 antagomirs. Moreover, the responsiveness to TGF-β ligand was fully restored as measured by the SMAD-responsive luciferase assay in vFLIP-transfected cells (Fig 4B). However, in vCyclin-transfected cells, SMAD2 protein level was fully restored while TGF-β responsiveness was partially restored (Fig 4C). Together, these results are consistent with reduction of SMAD2 protein levels by vFLIP and vCyclin being mediated via increased expression of miRNAs in the miR-17-92 cluster.

### SMAD2 inhibition was reduced in cells infected with KSHV mutants lacking vFLIP or vCyclin

We utilized mutant viruses, which do not express either vFLIP or vCyclin, in order to study the contribution of vFLIP or vCyclin to SMAD2 regulation in the context of a latent virus infection. To generate single or double knock-out mutant viruses of vFLIP or vCyclin, KSHV BAC16 was used [34]. LANA, vFLIP, vCyclin and viral miRNAs are all expressed from a single promoter, which gives rise to polycistronic multiply spliced mRNAs. To mutate vFLIP or vCyclin without affecting the complex RNA expression pattern in this locus, we mutated start codons rather than deleting open reading frames. After mutant bacmids were confirmed by sequencing, recombinant virus was recovered by first transfecting bacmid DNA into 293 cells followed by co-cultivation with iSLK cells.

**Fig 5:**
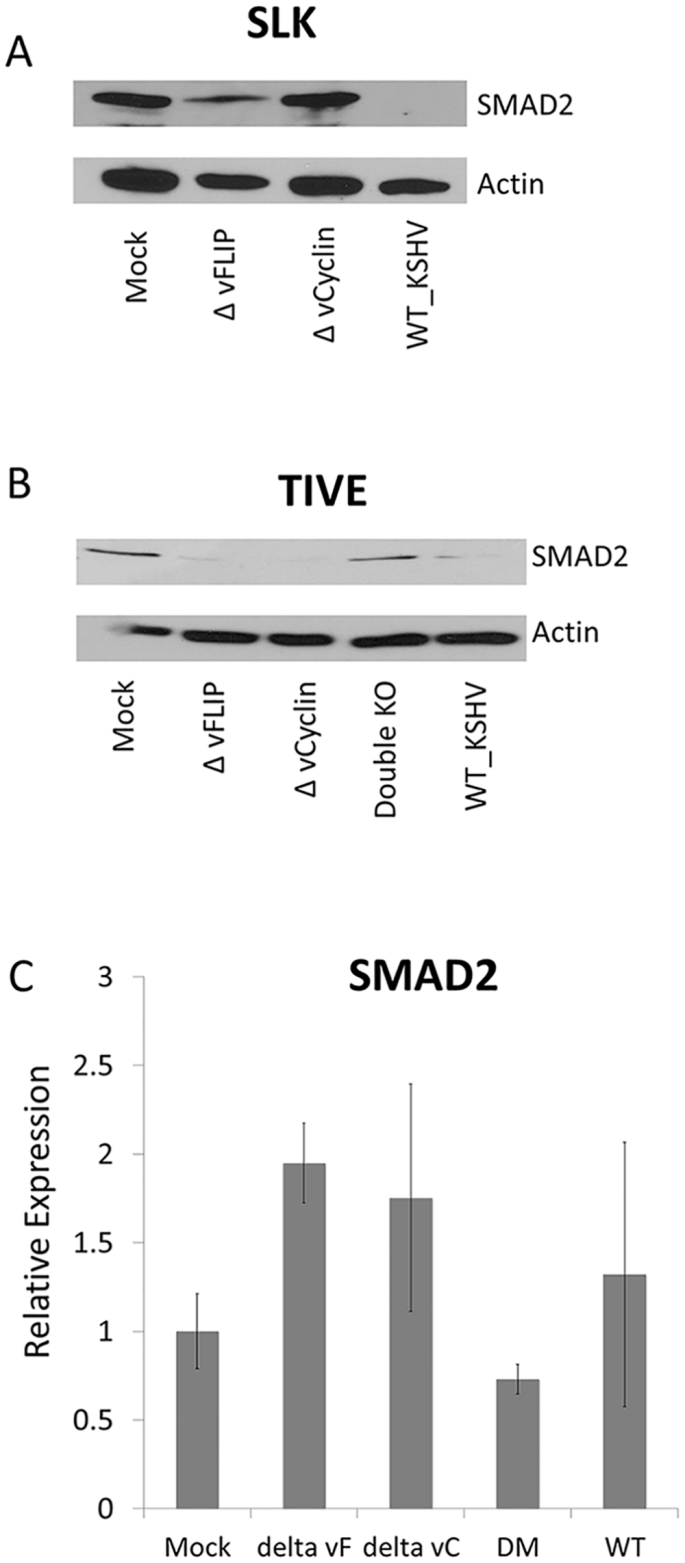
vFLIP or vCyclin deletion mutant virus recovered the SMAD2 expression in SLK or TIVE cells. (A and B) Western blot for SMAD2 in iSLK or TIVE cells infected with wild type KSHV or with mutant KSHV lacking vFLIP, vCyclin, or both. The infected cells were selected at least 4 weeks and harvested to detect SMAD2 expression. Actin was used as internal control. The experiments were performed twice independently, and one representative blot is shown. (C) qRT-PCR for SMAD2 and miR-17-92 from TIVE cells infected with wild type KSHV or with mutant KSVH lacking vFLIP, vCyclin and both. RNAs were harvested from latently infected TIVE cells and qRT-PCR was performed in triplicate. The expression of non-infected TIVE cell set at 1. GAPDH was used as internal control for normalization. The experiment was done three times independently.

Next we monitored SMAD2 expression in mock, vFLIP and vCyc single knockout, and WT-infected iSLK cells (Fig 5A). As previously seen in SLK cells, a high level of SMAD2 protein was detected in mock-infected iSLK cells, but was not detectable in WT-infected iSLK cells. Infection of iSLK cells with the ΔvFLIP mutant resulted in a detectable but significantly lower expression level of SMAD2. In contrast, infection with the ΔvCyclin mutant virus restored SMAD2 protein levels similar to that seen in uninfected iSLK cells. This indicates that in iSLK cells inhibition of SMAD2 by vCyclin is stronger than by vFLIP.

Since it is was recently demonstrated that SLK cells, long thought to be of endothelial origin, are actually are derived from an adenocarcinoma of epithelial origin [35], we also wanted to test vFLIP and vCyc-dependent regulation of SMAD2 in TIVE cells, an endothelial cell model in which to study KSHV pathogenesis [36]. Wt or mutant virus-infected iSLK cells, which express the RTA gene as an inducible transgene [37], were used to generate high titer virus that after quantification was used to stably infect TIVE cells. Cells were infected with 200 genome equivalents per cell and complete infection was confirmed by monitoring GFP expression, and subsequently, lysates and total RNA was collected for Western blot and RT-qPCR to monitor SMAD2 expression.

While the expression of SMAD2 in TIVE cells is lower than in SLK cells, WT KSHV infection decreased SMAD2 levels as observed in SLK cells. However, infection with either ΔvFLIP or ΔvCyc mutant viruses did not restore SMAD2 expression in TIVE cells suggesting differences by which both proteins contribute to the up-regulation of the miR-17-92 cluster between both cell types. To address this, a ΔvFLIP/ΔvCyc double knock-out mutant was generated as described above. Infection of TIVE cells with the ΔvFLIP/ΔvCyc mutant fully restored the SMAD2 expression to levels observed in uninfected TIVE cells (Fig 5B). Together, these data confirm that both KSHV vFLIP and vCyc regulate SMAD2 in the context of viral infection in cells of epithelial and endothelial origin, albeit at different efficiencies. While vFLIP or vCyclin expression alone is sufficient for down-regulation of SMAD2 in TIVE cells, both genes are required for inhibition of SMAD2 expression in SLK cells where base level SMAD expression is higher. Most miRNAs moderately modulate protein levels [38,39]. However, the observed down-regulation of SMAD2 by the miR-17-92 cluster was surprisingly strong.

To test the effects of miR-17-92 dependent targeting on SMAD2 mRNA turnover, real-time PCR was performed. Steady-state SMAD2 mRNA levels were not significantly changed in KSHV infected TIVE cells, compared to mock infected cells (Fig 5C). We observed a slight increase in SMAD2 mRNA in cells infected with single knock-out mutant. However, overall the significant decrease in SMAD2 protein, as detected by Western blotting, cannot be attributed to increased mRNA turnover. This indicates that the miR-17-92 cluster led to down-regulation of SMAD2 by mainly inhibiting translation. In summary, vFLIP and vCyclin contribute to inhibition of TGF-β signaling by transcriptionally activating the miR-17-92 cluster, which results in decreased translation of SMAD2 mRNA.

## Discussion

A number of KSHV latency-associated genes modulate the TGF-β signaling pathway. Firstly, KSHV-encoded miRNAs modulate directly or indirectly TGF-β signaling by targeting TGFBR2, SMAD5, and THBS-1 [24,40,41]. Secondly, LANA directly down-modulates the TGF-β receptor in PEL and endothelial cells [25]. Here, we report a third way to target TGF-β signaling by inducing host miRNA expression. vFLIP and vCyc promote increased expression of the miR-17-92 cluster, which targets SMAD2 protein synthesis via miR-17-5p, miR-18a, and miR-20. Complete loss of SMAD2 was not only observed in SLK cells over-expressing vFLIP and vCyc by transfection, but also in WT KSHV infected cells of both epithelial (SLK) and endothelial (TIVE) origin (Figs 3A, 5A and 5B). Interestingly, the individual contributions of vFLIP and vCyc to this regulatory loop were different in the two cell types. In latently infected SLK cells, deletion of vCyc was sufficient to restore SMAD2 expression to nearly wt levels whereas deletion of vFLIP did not restore SMAD2. Moreover, the rescued response to TGF-β in the presence of antagomirs was lower in vCyc-, compared to vFLIP-transfected cells (Figs 4B, 4C and 5A). In TIVE cells, which express lower levels of SMAD2, expression of either vFLIP or vCyc alone was sufficient to cause down regulation of SMAD2, and only a double knockout restored expression to wt levels (Fig 5B).

Using reporter assays and RT-PCR we demonstrate transcriptional upregulation of the miR-17-92 cluster, but how these viral genes induce this promoter has not been fully resolved. Overexpression of the miR-17-92 cluster in a Myc transgenic mouse model accelerated malignant lymphoma growth and provided the first evidence of miRNA oncogene activity [42]. We note that KSHV also de-regulates the oncomir miR-155 by either inducing miR-155 or expressing a viral ortholog miR-K12-11 (reviewed in [43]). In addition, c-Myc, a gene dys-regulated in many cancers, can transcriptionally activate the miR-17-92 promoter [18,42]. Activation of Myc has been observed in latently infected PEL cells, where it contributes to maintenance of latency [44]; however, Myc was not detectable in SLK cells by Western blot analysis (S1A Fig), indicating that in these cells miR-17-92 is not induced via Myc activation.

Sylvestre et al. reported that the transcription factor E2F1 augments transcription from the miR-17-92 promoter. Furthermore, miR-20a, a member of the miR-17-92 cluster, transcriptionally down-regulated E2F2 and E2F3 but not E2F1 [19]. Therefore, it is plausible that vCyc, an ortholog of cellular cyclinD, activates miR-17-92 expression in part through this auto-regulatory feedback loop, by inducing the E2F family transcription factors [11,45].

vFLIP is a potent activator of NF-κB signaling which is required for PEL cell survival [4]. Moreover, vFLIP down-regulates CXCR4 by miR-146a in a NF-κB dependent manner [46]. Epstein-Barr virus (EBV), a γ-herpesvirus associated with multiple malignancies, also induces several host miRNAs by LMP1 in an NF-κB dependent manner [47,48,49,50,51]. Moreover, vFLIP-induced NF-kB, also drives miR-146a expression in PEL cells. Based on these similarities, we tested if the activation of the miR-17-92 cluster by vFLIP was NF-kB-dependent. Even under conditions where NF-kB signaling was blocked by the inhibitor, Bay-11 (S1B Fig), the expression of the miR-17-92 cluster was still induced by vFLIP (S1C Fig) indicating the involvement of another signaling pathway. Interestingly, not all viral FLIP proteins activate NF-kB. Molluscum Contagiosum Virus (MCV) is a poxvirus that encodes two FLIP proteins termed MC159 and MC160, which encode two DED domains but contrary to KSHV vFLIP do not induce NFkB but rather inhibit it [52]. We therefore asked whether the novel activity of vFLIP to augment transcription from the 17/92 promoter is conserved in the MCV FLIP proteins. In co-transfection experiments the 17/92 promoter was induced between 2.5- and 5.5-fold by MC159 and MC160, respectively (S2 Fig). Hence, in addition to inhibiting apoptosis, modulating NFkB positively or negatively, and inhibiting IRF-3, viral FLIP proteins also induce the oncogenic miR-17-92 cluster. MC159 and MC160 activate the 17/92 promoter without inducing NF-kB, suggesting that KSHV vFLIP may not require NF-kB activation to induce miR-17-92 expression.

Targeting TGF-β signaling in endothelial cells via a number of different mechanisms underlines the importance of this pathway for KS sarcomagenesis. With respect to different epithelial cell tumors many reports have described a so called “TGF-β paradox” in that during the early stages of tumorigenesis TGF-β is blunted to protect cells from apoptosis, while at later stages this pathway is severely activated [53,54]. Moreover, it has recently become clear that TGF-β in endothelial cells regulates angiogenesis, a hallmark of KS tumors, either positively or negatively (reviewed in [55]). At high levels of TGF-β, signaling occurs through phosphorylation of SMAD2, 3, and 4, which in endothelial cells is antiangiogenic and in fully transformed cells is associated with NF-kB activation. Conversely, at low concentrations of TGF-β, signaling occurs through phosphorylation of SMAD 1,5, and 8 which is proangiogenic and induces proliferation and migration associated with high levels of ID1 known to be activated in KSHV latently infected cells [56,57]. Interestingly, similar opposing activities with respect to angiogenesis have recently been identified for components of the miR-17-92 cluster, although collectively expression of the miR-17-92 miRNA cluster is proangiogenic. Two 17/92 cluster miRs, miR18a/19 target the antiangiogenic secreted factor Tsp-1, thereby promoting angiogenesis in the tumor environment. Conversely miR17/20 and miR-92a target Janus kinase 1 (Jak1) and integrin a5 (Itga5) which negatively regulates vascular morphogenesis (reviewed in [55,58,59]).

Based on our new data and previously published data on LANA and viral miRNAs [24,25], we propose a model whereby KSHV latently infected cells target the TGF-β pathway and the miR17/92 cluster to protect cells from apoptosis and at the same time regulate angiogenesis through autocrine and paracrine mechanisms in both the tumor and its microenvironment. Interestingly, viral and virally-induced host miRNAs can reinforce TGF-β signal outcome to support angiogenesis by targeting SMAD2 and Tsp-1. Finally, LANA’s ability to reduce TGFBR expression at the cell surface further reduces the sensitivity of latently infected cells to TGF-β. In summary, we identified new activities for vCyc and vFLIP which via the induction of the miR17/92 cluster integrate three different viral mechanisms to promote angiogenesis via TGF-β signaling.

## Materials and Methods

### Cell lines

293T (American Type Culture Collection), SLK, and iSLK cells (NIH AIDS Research and Reagent Program), were cultured in DMEM with 10% FBS and 1% penicillin and streptomycin. Telomerase immortalized vein endothelial cells (TIVE) and long-term cultured KSHV infected cells (TIVE-LTC) have been generated in our laboratory and have been previously described [36]. TIVE cells were cultured in Medium199 with 60 μg/mL of endothelial cell growth supplement (Sigma), 20% FBS and 1% penicillin and streptomycin. iSLK or TIVE cells, infected with KSHV BAC16 [34] wild type or mutant viruses, were treated with 50 μg/mL Hygromycin for maintaining latently infected cells. TGF-β ligand was purchased from AbCam (Cat#ab50036).

### MiRNA expression profiling

Total RNA was extracted from cells using RNA-Bee reagent (Tel-Test, Inc. TX), and quality and yields analyzed using Agilent Bioanalyzer and Nanodrop. RNAs were labeled using the miRCURY LNA microRNA Array Labeling kit (Exiqon). 3’-ends of the total RNA were enzymatically labeled with the Hy3 fluorescent dye (Exiqon) using T4 RNA ligase. Labeled RNA was hybridized to the LMT_miRNA_v2 microarray, which was designed using the Sanger miR9.0 database (http://microrna.sanger.ac.uk) and custom manufactured by Agilent Technologies as 8 x 15k microarrays. 1667 unique mature miRNA sequences across all species were incorporated into 60-mer oligonucleotide probes with a 3’ linker sequence to allow separation from the glass slide surface. The Agilent linker sequence has minimal homology to any GenBank sequence. Each mature miRNA is represented by + and–(reverse complement) strand sequences, and each probe has 4 replicates within each microarray, giving 8 probes per unique mature miRNA. 10 sets of random 22mer sequences served as negative controls. Positive (normalization) controls were designed using U1, U2, U4, U5 and U6 sequences. 22mer sequences corresponding to 5’ and 3’ ends of the small nuclear RNAs were incorporated into 60 mer probes (a total of 8 x 5 x 2 x 2 = 160 probes). Additional controls such as probes to Actin, GAPDH, HSP70 and Line elements are present on the microarray. In total 3556 unique LMT seq ids (miRNA, positive and negative controls, +/- strand) were on the microarray.

A 2x Hybridization Buffer and 10x blocking buffer (Agilent) were added to the fluorescently labeled miRNAs. The samples were heated to 99°C for 3 minutes and snap-cooled before being added to the microarray printed on glass slides and hybridized for 16 h at 47°C. The glass slides were washed with the Agilent wash buffer 1 (room temperature) and 2 (at 37°C), dried with the Agilent stabilization and drying solution, and scanned using the Agilent scanner (model G2505B). The Agilent Scan Control software (version A.7.0.3) was used to produce a high resolution tif image file.

The Agilent Feature Extraction Program (FEP), version 9.5.3.1, was used to identify feature spots and extract signal intensity values. Two types of signal intensity data were used in subsequent analysis: the raw mean signal intensity of the green channel pixels in each feature spot (gMeanSignal) and the average local background signal intensity of the pixels relative to the feature spot (gBGMeanSignal). To compensate for artifacts introduced by outlier background signal intensity values due to the features position on the array, a perl script calculated the average gBGSignal for all features on the chip at the same position on the array (eight arrays per chip). The script then subtracted the average gBGSignal from each replicate feature spot signal intensity (gMeanSignal) and then calculated the average of all background subtracted replicate features per array.

### Plasmids

The expression constructs for LANA (pcDNA3.1/LANA) and KSHV cluster miRNAs (pcDNA3.1/cluster) were described in previous reports [24,33]. For construction of vFLIP and vCyclin expression vectors, Gateway Cloning method was used. After ORFs of vFLIP and vCyclin were amplified by PCR and cloned into entry vector (pDONR222, Invitrogen) using BP recombination, ORFs were cloned into pLenti6/V5-DEST (Invitrogen) using LR recombination following the manufacturer’s procedure to create pLenti6/vFLIP and pLenti6/vCyclin. Reporter vector containing the promoter of miR-17-92 cluster upstream of luciferase vector was kindly provided from Dr. De Guire [19]. pGL3-SBE4 (Promega) contains four SMAD binding elements upstream of luciferase gene, activated by TGF-β ligand. pCMV-Renilla (Promega), expressing renilla luciferase, was used for normalization of firefly luciferase activity.

### Transfection and luciferase reporter assays

For transfection of latently infected SLK cells, electroporation (NucleofactorII, Amexa) was used with Kit V following the manufacturer’s protocol. 10^6^ of SLK or KSHV infected SLK cells were used for each transfection. Antagomirs of miRNAs, miR-17, 18a, and 20, (Dharmacon, CO) were co-transfected with plasmids using the same methods. For luciferase assays and Western blot analysis, SLK cells were seeded 24 hours prior to transfection and transfected using TransIT-293 reagent (Mirus, WI) at 2.5 x 10^5^ cells per well for 6-well plates or 0.5 x 10^5^ cells per well for 24-well plates, according to the manufacturer’s protocol. Firefly luciferase activity was quantified using the Dual Luciferase Reporter kit (Promega, WI) according to the manufacturer’s protocol. 0.5 μg of reporter vectors (pGL3/PmiR-17-92 and pGL3-SBE4) were co-transfected with expression vectors of latent genes (LANA, KSHV miRNA, vFLIP and vCyclin), and harvested 48 hrs after transfection. Antagomirs were transfected together with reporter vectors and expression vectors. 2 ng/mL of TGF-β ligand was added at 24 hours post-transfection and the cells were harvested at 72 hours post-transfection. 2 ng of the pCMV-Renilla (Promega, WI) was used for luciferase analysis. FLUOstar OPTIMA reader (BMG Labtech) was utilized for measuring firefly luciferase activity, which was normalized to Renilla luciferase activity. All assays were performed as three independent experiments and standard deviation was calculated for triplicates and displayed as error bars.

### Western blot analysis

Cells were harvested 48 hours after transfection or treatment with TGF-β ligand and lysed in lysis buffer (20mM HEPES, 100mM KCl, 0.2mM EDTA, 0.5mM DTT, 2.5% Glycerol, and protease inhibitor (Roche)). 5–10 μg of total protein were loaded in each lane of 10% SDS PAGE gels and transferred to PVDF membranes. Anti-V5-HRP antibody (Invitrogen,CA, 46–0708) was utilized to detect and confirm V5-tagged vFLIP and vCyclin expression from plasmid constructs. Primary antibody for detecting SMAD2 was purchased from Cell Signaling Technology Inc. (Beverly, MA, Cat #3103).

### Quantitative reverse transcription-PCR (RT-qPCR) analysis

RNA-Bee (Tel-Test, TX) was utilized to extract RNAs from TIVE cells according to the manufacturer’s instructions. SuperScript III (Invitrogen, CA) was used to synthesize cDNA according to the manufacturer’s procedure. Quantitative RT-PCR (qRT-PCR) analysis was carried out using an ABI StepOne Plus system (Applied Biosystems, CA). GAPDH was used as internal control to normalize the expression of all genes. Student t-tests were performed for statistical significance compared to non-infected cells.

### Generation of BAC16-derived recombinant KSHV bacmids in *E*. *coli*

Mutants were constructed as described by Brulois et al. using the BAC16 backbone [34]. In brief, the individual start codon ATG was mutated to TCG for vCyclin and vFLIP, whereas in the double mutant both start codons were changed to TCG by using two step red recombineering. The Kan/I-sceI cassette was generated by using primers containing flanking regions and Kan/I-sceI amplification primers. The gel purified linear cassette was electroporated into freshly red activated (42°C) electrocompetent *E*.*coli GS1783* cells harboring BAC16 to carry out intermolecular recombination. The transformants were grown on Kan containing LB media overnight at 30°C. After the verification of Kan insert and bacmid integrity using colony PCR and PFGE respectively, the second Red intramolecular recombination was carried out by activating arabinose inducible SceI system and temperature sensitive Red recombination system. The marker-less mutants were verified for bacmid integrity using PFGE and were sequenced using Sanger sequencing for the confirmation of replacements in all three mutants.

### Recovery of infectious recombinant KSHV in iSLK cells

After quality control, wt and mutant bacmids were initially transfected into 293T cells and selected with hygromycin. Two weeks later 293T cells were induced with TPA and co-cultured with iSLK cells that contain the RTA transcactivator as an inducible transgene [37]. After 72 hours co-cultures were treated with puromycin and hygromycin to select KSHV-infected iSLK cells and to kill off residual 293T cells. Two weeks after cultivation iSLK cells were 100% GFP-positive. Subsequently, iSLK cells infected with wild type or delta vFLIP, vCyc or double mutant were induced with 1 μg/mL doxycycline and 1 mM NaB for 72 hours. The media supernatant was passed through a 0.45 μM filter virus particles were centrifuged at 100,000 x g for 1 hour. The number of virus particles was quantified by qPCR assay after viral DNA extraction using DNAzol (Molecular Research Center, Inc.). Serially diluted LANA expression plasmid was used as standard curve. Resulting virus was used to infect TIVE cells using 100 genome equivalents per cell, which yields 100% GFP positive cells 48 hrs post infection.

## Supporting Information

S1 Fig Induction of miR-17-92 is independent of myc and NF-κB. (A) SLK cells do not express cellular Myc. Western blot for c-Myc was performed with SLK cells transfected with empty vector or with vFLIP- or vCyclin-expressing vectors. Cells were harvested 48 hour post-transfection. The positive control is the lysate of T-lymphoblastic leukemia cell, KOPT-K1 cell. Actin was used as internal control. (B and C) The miR-17-92 cluster expression induced by vFLIP and vCyclin is not decreased by the NF-κB inhibitor Bay-11. (B) Activation of the NF-κB reporter by vFLIP in the presence of the NF-κB inhibitor Bay-11 was assessed by transfecting with an NF-κB-firefly luciferase construct. Firefly and renilla luciferase activities were measured, and firefly luciferase activity was normalized to control renilla luciferase activity. Addition of Bay-11 at 2, 4 or 6 μM resulted in decreased luciferase activity, confirming that the inhibitor was active in these cells at these concentrations. (C) Luciferase reporter analysis was performed with reporter vector containing the promoter of the miR-17-92 cluster, co-transfected with vector expressing either vFLIP or vCyclin in the presence of various concentrations of Bay-11. The cells were harvested 48 hours after transfection. Results were normalized to the control cells treated with DMSO instead of Bay-11. Bay-11 did not decrease activation of the miR-17-92 promoter by vFLIP or vCyclin.
(TIF)

S2 Fig MCV FLIP proteins, MC159 and MC160, activate the miR-17-92 promoter. MC159 and MC160 expression vectors were co-transfected with reporter vector (pGL3-PmiR-17-92) into SLK cells and firefly luciferase activity measured and normalized as above.
(TIF)

S1 Table Primer sequences. (DOCX)
