# Supplementary figures and images for: Kaposi’s Sarcoma-Associated Herpesvirus (KSHV) Induces the Oncogenic miR-17-92 Cluster and Down-Regulates TGF-β Signaling
Source: PLoS Pathog. 2015 Nov 6;11(11):e1005255. doi: 10.1371/journal.ppat.1005255 (PMC4636184; doi:10.1371/journal.ppat.1005255)

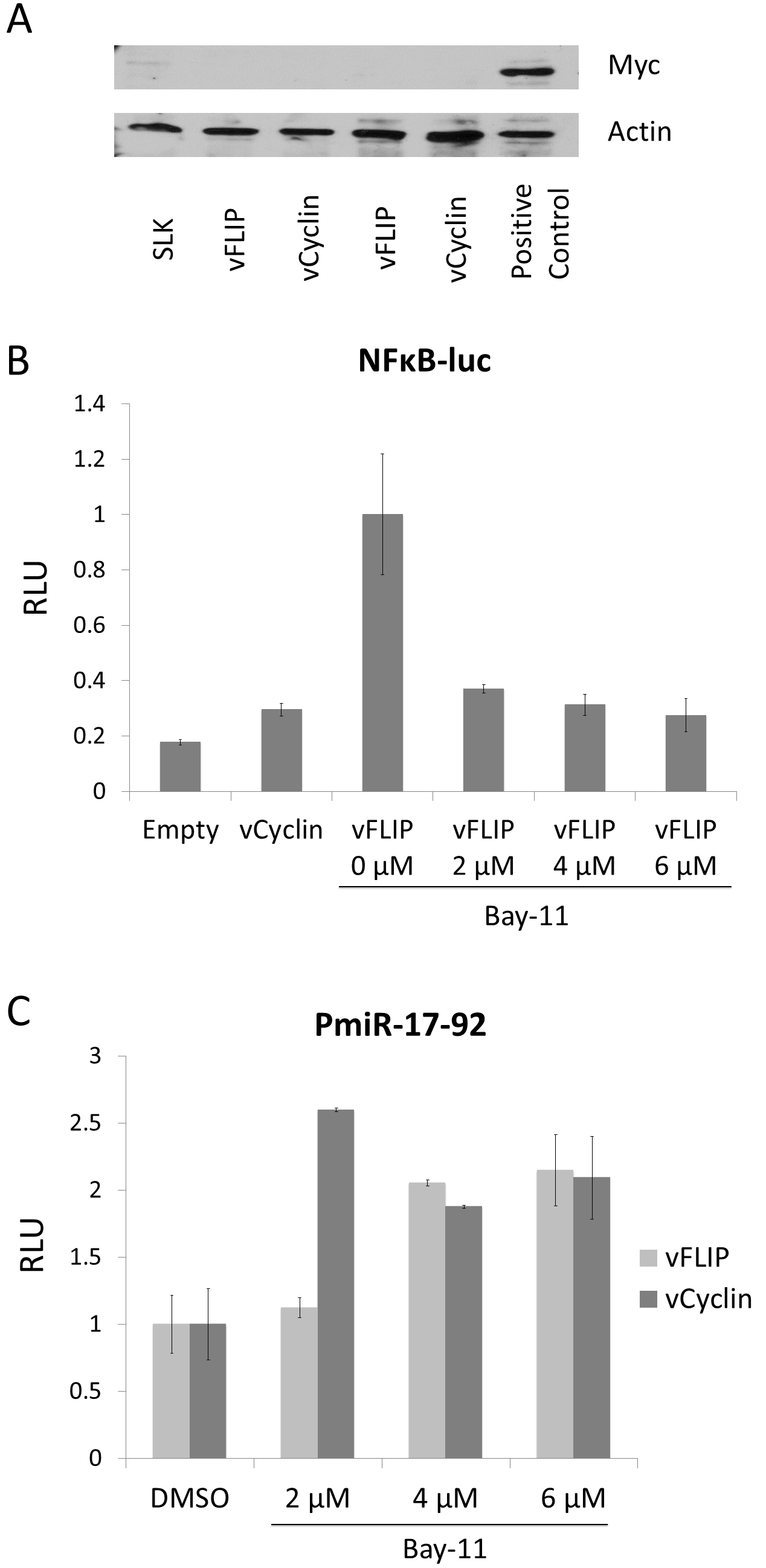

Supplement: S1 Fig — (A) SLK cells do not express cellular Myc. Western blot for c-Myc was performed with SLK cells transfected with empty vector or with vFLIP- or vCyclin-expressing vectors. Cells were harvested 48 hour post-transfection. The positive control is the lysate of T-lymphoblastic leukemia cell, KOPT-K1 cell. Actin was used as internal control. (B and C) The miR-17-92 cluster expression induced by vFLIP and vCyclin is not decreased by the NF-κB inhibitor Bay-11. (B) Activation of the NF-κB reporter by vFLIP in the presence of the NF-κB inhibitor Bay-11 was assessed by transfecting with an NF-κB-firefly luciferase construct. Firefly and renilla luciferase activities were measured, and firefly luciferase activity was normalized to control renilla luciferase activity. Addition of Bay-11 at 2, 4 or 6 μM resulted in decreased luciferase activity, confirming that the inhibitor was active in these cells at these concentrations. (C) Luciferase reporter analysis was performed with reporter vector containing the promoter of the miR-17-92 cluster, co-transfected with vector expressing either vFLIP or vCyclin in the presence of various concentrations of Bay-11. The cells were harvested 48 hours after transfection. Results were normalized to the control cells treated with DMSO instead of Bay-11. Bay-11 did not decrease activation of the miR-17-92 promoter by vFLIP or vCyclin. (TIF) [file ppat.1005255.s001.tif]

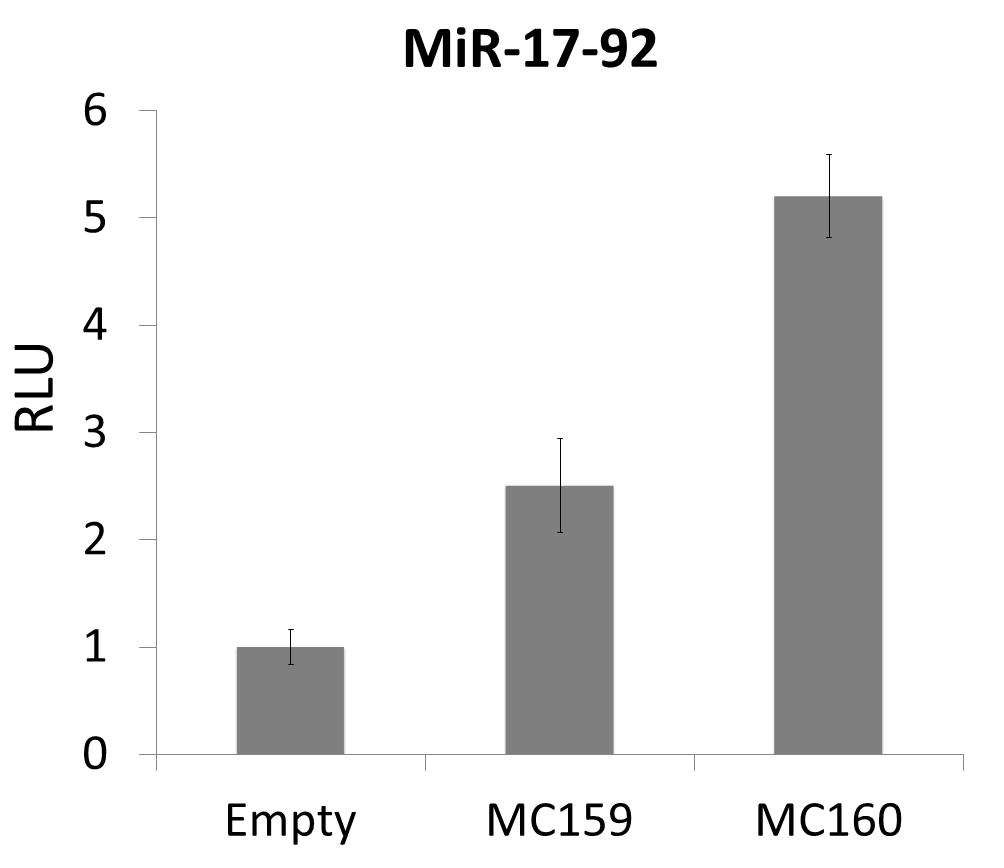

Supplement: S2 Fig — MC159 and MC160 expression vectors were co-transfected with reporter vector (pGL3-PmiR-17-92) into SLK cells and firefly luciferase activity measured and normalized as above. (TIF) [file ppat.1005255.s002.tif]
