# Supplementary material for: Kaposi’s Sarcoma-Associated Herpesvirus (KSHV) Induces the Oncogenic miR-17-92 Cluster and Down-Regulates TGF-β Signaling
Source: PLoS Pathog. 2015 Nov 6;11(11):e1005255. doi: 10.1371/journal.ppat.1005255 (PMC4636184; doi:10.1371/journal.ppat.1005255)
Supplement: S1 Table — (DOCX) [file ppat.1005255.s003.docx]

**S1 Table. Primer sequences**

| Primer name | Sequence |
| --- | --- |
| vFLIP Forward | 5' AAAGCAGGCTCCACCATGGCCACTTACGAGGTTCTC 3’ |
| vFLIP Reverse | 5’ ACAAGAAAGCTGGGTCCAATGGTGTATGGCGATAGTG 3’ |
| vCyclin Forward | 5' AAAGCAGGCTCCACCATGGCAACTGCCAATAACCC 3’ |
| vCyclin Reverse | 5’ ACAAGAAAGCTGGGTCCAAATAGCTGTCCAGAATGCG 3’ |
| miR-17-92 F | 5’ TGTGGTAGTGAAAAGTCTG 3’ |
| miR-17-92 R | 5’ AAATCTTCTGGTCACAATCC 3’ |
| SMAD2 F | 5’ GTGCCTAAGTGATAGTGC 3’ |
| SMAD2 R | 5’ GTACTTGTTACCGTCTGC 3’ |
| GM82 | 5′ GGGGACCACTTTGTACAAGAAAGCTGG GTC 3’ |
| GM81 | 5′ GGGGACAAGTTTGTACAAAAAAGCAGGCTCC 3’ |
| GAPDH F | 5′ CCCCTGGCCAAGGTCATCCA 3′ |
| GAPDH R | 5′ ACAGCCTTGGCAGCGCCAGT 3′ |
